# Hybridization Resulted in Shifts from Dioecy to Monoecy in Weeping Willows (*Salix* L.)

**DOI:** 10.3390/genes16080958

**Published:** 2025-08-13

**Authors:** Pablo Alarcón-Bolaños, Loïc Pittet, Li He, Elvira Hörandl

**Affiliations:** 1 Department of Systematics, Biodiversity and Evolution of Plants (with Herbarium), University of Göttingen, 37073 Göttingen, Germany; p.alarconbolanos@stud.uni-goettingen.de (P.A.-B.); loic.pittet@biologie.uni-goettingen.de (L.P.); 2 Eastern China Conservation Centre for Wild Endangered Plant Resources, Shanghai Chenshan Botanical Garden, Shanghai 201602, China

**Keywords:** hybrids, genetic structure, plant cultivation, RADSeq, sex determination systems

## Abstract

Background/Objectives: In flowering plants, hybridization is an important evolutionary force that might change sex distributions and sex determination systems (SDSs). However, little is known about processes in the first hybrid generations. Here, we study a cultivated putative hybrid cross of weeping willows (genus *Salix*, *S*.), *S. alba* × *babylonica* to gain insights into the effects of hybridization into SDSs. Methods: We analyzed the genetic structure of pure *S. alba*, pure *S. babylonica*, and the putative hybrid crosses in Central Europe using RADSeq data and five independent methods (NeighborNet, genetic structure analysis, Principal Component Analysis, hybrid index and heterozygosity analysis, and hybrid class analysis). The genetic SDS was analyzed on male, female, and mixed (monoecious) phenotypes by detecting sex-specific genomic markers using RADSex. Results: Genetic analyses indicate that most of the weeping willows represent F1 hybrids (*S. alba* × *babylonica*), and only two putative *S. alba* backcrosses. Hybrid index, heterozygosity, and hybrid class analyses provided more interpretable results than the other methods. The parental species were consistently dioecious, whereas hybrids had male, female, and monoecious phenotypes. RADSex revealed a male heterogametic XY system for *S. alba*, and this was combined in the hybrids with the previously known ZW system of *S. babylonica*. Conclusions: We confirmed the historical records stating that *S. alba* × *babylonica* are mostly F1 hybrids. We report for the first time that the combination of XY and ZW systems in primary hybrids results in regular shifts to monoecy.

## 1. Introduction

Hybridization between species is a major evolutionary force for flowering plants, which may result in novel genotypes, introgression, or even in hybrid speciation [1,2,3,4,5]. Hybrid fitness in plants is highly variable, and genotypes with some fertility will be able to reproduce beyond the first generation, potentially resulting in backcrosses with the parents, hybrid zones, or even the formation of new hybrid lineages [6,7]. Hybridization may produce vigorous highly heterozygous genotypes in the F1 generation that benefit from heterosis effects. Plant breeders often produce artificial hybrids to achieve novel, vigorous phenotypes and genotypes with certain character combinations [8,9,10,11].

Hybrids may further express altered sex distributions compared to their parents. While most flowering plants have hermaphroditic flowers (c. 72% of species), c. 5% are monoecious, i.e., they bear male and female flowers on the same individual; about 5–6% of plants species are dioecious, i.e., individuals bear either male or female flowers; the remaining percentages belong to other mixed co-sexual systems [12,13,14]. In dioecious plants, sex determination is usually controlled by one- or two-gene systems acting within a sex-linked region on one chromosome, which is usually small, not degenerated, and mostly morphologically similar to its counterpart [15,16]. Dioecious plants may have either male heterogametic (XY) or female heterogametic (ZW) systems, and in some genera (*Populus*, *Salix*) both systems may occur in the same genus [17,18,19,20,21]. Shifts from dioecy to various forms of co-sexuality has occurred in lineages, but the genetic and evolutionary backgrounds of such shifts is still not well understood, as most cases are known from targeted plant breeding [16]. Some shifts from dioecy to co-sexuality could have originated from hybridization in combination with polyploidization (allopolyploidy), e.g., in *Mercurialis* [22]. However, polyploidization by itself imposes constraints against dioecy [23], and little is known about the processes directly after homoploid hybridization (i.e., with parents and hybrids having the same ploidy level). In homoploid hybrids, different sex determination systems of parents (XY/ZW) were thought to be a strong crossing barrier between species [18]. In general, little is known about the immediate impact of hybridization on sex determination in plants.

The genus *Salix* L. (=*S.*, willows) is an appropriate model system for studying the impact of hybridization on sex determination systems (SDSs). The genus comprises trees and shrubs, and is distributed in the Northern Hemisphere and in South America, with c. 400–500 species [24,25]. All species are dioecious, with flowers united in elongated inflorescences called catkins; only very rarely does dioecy appear unstable in natural populations, with sex liability restricted to few individuals [26]. Both XY and ZW sex determination systems occur in different species of the genus, and the sex-linked regions reside on different chromosomes [18,20]. Hybridization is common within the genus in natural populations [7,27,28,29,30,31], but hybrids have also been produced by artificial crosses and used for cultivation [11,29,32]. Various willow species and their hybrids have been cultivated in Europe since the time of Roman empire for, e.g., stabilization of riversides and wet slopes, as windbreakers, for basketry, as medicinal plant as source of salicylic acid, and as ornamentals [11,32,33].

**Figure 1:**
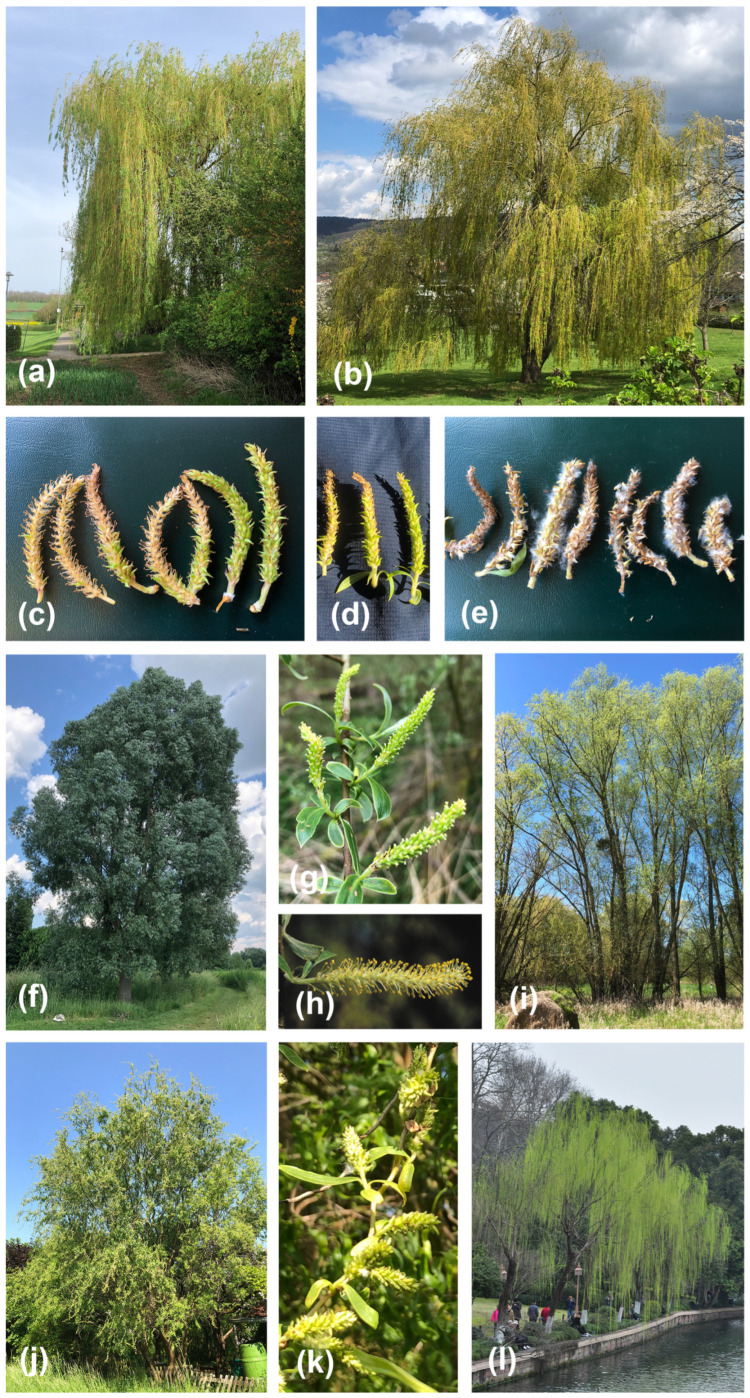
Phenotypes of willows (*Salix = S.*); *S. alba × babylonica* (**a**–**e**), *S. alba* (**f**–**i**), and *S. babylonica* (**j**–**l**). (**a**) Habit of a weeping willow (EH11381); (**b**) habit of weeping willow (EH11230); (**c**) catkins of EH11381 during anthesis, with two male catkins on the left, three mixed male–female ones in the middle, and two female catkins on the right; (**d**) catkins of weeping willow (EH11230) during anthesis, two mixed male–female ones (left) and one female (right); (**e**) catkins of weeping willow (EH11230) at fruiting stage (one male on the left, four females with seeds on the right); (**f**) habit of *S. alba* (EH11279, after anthesis, with silvery leaves); (**g**) female catkins of *S. alba* (EH11247); (**h**) male catkin of *S. alba* (EH11279); (**i**) habit of *S. alba “excelsa”* (EH_11235); (**j**) habit of *S. babylonica “tortuosa”* (EH11264); (**k**) fruiting female catkins of *S. babylonica* (EH11264); (**l**) Habit of *S. babylonica* (Hangzhou). Photo credits: (**a**–**k**), E. Hörandl; (**l**), L. He.

One of the most popular ornamental trees in Europe is the “weeping willow” which are thought to be artificial hybrids between *Salix alba* s.l. and *S. babylonica*, and are characterized by conspicuous pendulous branches (Figure 1a,b). Both parental species belong to the same subclade in the genus, which has been classified as *Salix* subg. *Salix* [5,25]. *S. alba* is a big tree native to Eurasia, characterizing the vegetation on riversides; the species has many varieties that have been used for various crossing purposes [11,32]. *S. alba* is of allotetraploid origin [34,35]. *S. babylonica* is a middle-sized tree with mostly pendulous branches, native to China [25,36]; in Europe it has been cultivated for c. 300 years, but only in the southern regions because of its frost sensitivity [32]. Recently, genome sequencing of native *S. babylonica* from China revealed it as an allotetraploid that originated some millions of years ago from diploid XY and ZW parental species, and established finally a female heterogametic (ZW) SDS on chromosome 15 [20]. The species was replaced in Central and Northern Europe by the artificial hybrid *S. alba × babylonica*, reported to be produced since the late 19th century, and planted in parks, gardens, and in cemeteries [11]. Interestingly, the hybrid bears male and female catkins on the same individual [32]. Since these hybrids must be primary or early generation hybrids, they offer the opportunity to study the background of shifts between dioecy and monoecy following homoploid hybridization on adult trees. Monoecy could be the result of different SDSs of parents, and eventually a transition phase to evolutionary SDS turnovers.

Willow species usually exhibit a high phenotypic variability between individuals and change characters during development from the anthesis to fruiting stage; therefore, hybrid identification based on morphology is only often misleading, and should be supported by analysis of molecular markers [27,29,30,31,37]. A recent population genetic study using DArTSeq markers on various willow hybrids [29] detected one pure *S. babylonica* tree cultivated in Czech Republic, and genetic structure analyses suggest *alba × babylonica* hybrids or backcrosses to *S. alba*. This raises the question whether *S. babylonica* grows more frequently in Central Europe and hybridizes in situ, and whether introgression with *S. alba* populations takes place. Vašut et al. [29], however, included just a few samples and had its focus on other willow hybrid combinations; sex distributions were not addressed. For the detection of hybrids, even of closely related species, genomic markers derived from restriction-site-associated markers (RADSeq) have been proved to be highly informative and cost-efficient for larger sets of samples [31,38,39,40,41,42]. Furthermore, the recently launched program RADSex [43] offers tools to test SDSs based on RADSeq data [44].

The objective of this research is to test whether weeping willows in Central Europe are actually hybrids of *S. alba* and *S. babylonica* and not pure *S. babylonica*. We want to analyze whether these trees represent F1 hybrids, later generation hybrids, or introgressants of *S. alba*. We further ask whether the hybrids are consistently monoecious. Finally, we want to test whether RADSeq data are informative about the genetic background of sex distributions and SDSs by analyzing molecular markers of male, female, and monoecious trees.

## 2. Materials and Methods

### 2.1. Sampling and DNA Extraction

We sampled branchlets, leaf material, and catkins from 42 individuals from *Salix* (=*S*.) *S. alba* (21 males and 21 females) from natural sites in Central Germany, 25 individuals from cultivated weeping willows (*S. alba × babylonica*), and 7 individuals from pure *S. babylonica* (one cultivated in C. Germany, two cultivated ones from Sicily, and four from China). Determination followed diagnostic characters of the taxa [11,32,33,36,45,46], summarized in Appendix A. Most weeping willows matched the “golden weeping willow” variety, *S. alba* var. *vitellina × babylonica* (=*S. × sepulcralis* Simonkai var. *chrysocoma* (Dode) Meikle) [32], but for simplicity and different taxonomic opinions on varieties [25] we name all our samples just *S. alba × babylonica* (sensu lato). Leaves were dried in silica gel for DNA analysis. For the assessment of sex, we examined the whole tree and sampled at least 10 representative catkins to determine whether they had male, female, or both flowers. Since the weeping willow trees were proterandric, with male catkins appearing in late March/beginning of April (and then rapidly withering and falling off), and female catkins flowering in late April until the end of May, many trees were visited twice to ensure monoecy versus male/female sex. Some trees produced male, female, and mixed (androgynous) catkins (Figure 1c,d). All samples, their sexes, and collection data are summarized in Supplementary Table S1, and a map of locations is provided in Supplementary Figure S1. Herbarium vouchers for all specimens are deposited in the herbarium of the University of Göttingen (GOET). The tetraploid level for *S. alba × babylonica* reported previously [47] was confirmed by flow cytometry (Supplementary Table S2) following methods as described [48].

### 2.2. DNA Extraction, RAD-Seq, and RAD-Loci Assembly

Genomic DNA was extracted from silica-dried leaf tissue using the DNeasy Plant Mini Kit (Qiagen, Hilden, Germany), following a modified version of the manufacturer’s protocol [30]. DNA concentrations were measured using a Qubit 3.0 fluorometer (Thermo Fisher Scientific, Waltham, MA, USA), and samples were normalized to 30 ng/μL prior to library preparation. After quality control, DNA extracts were sent to Floragenex Inc. (Beaverton, OR, USA) for single-end RAD-seq library preparation and sequencing. Library construction followed the protocol described by [49] and involved digestion with the restriction enzyme PstI and fragment size selection between 300 and 500 bp. Sequencing adaptors and unique 10 bp barcodes were ligated to each sample. The libraries were then sequenced on an Illumina NovaSeq6000 platform (Illumina Inc., San Diego, CA, USA), and the resulting raw reads were delivered in FASTQ format.

The RAD-loci assembly was performed using the Stacks software pipeline v.2.68 [50]. Reads were demultiplexed, filtered for low quality, and trimmed to 128 bp using the program process_radtags. Sample quality was then assessed first with FastQC v.0.11.4 [51] and subsequently with MultiQC v.1.27.1 [52] to generate a quality report for all samples. The denovo_map.pl program from the Stacks pipeline was executed first to explore the optimum assembly parameters using the R80 method [53] by adjusting the parameters -M (maximum number of mismatches between stacks within individuals) and -n (maximum number of mismatches between stacks between individuals) to assess changes in loci and identify the best M/n combination for this dataset. The optimal combination was achieved with a value of 2 for both -M and -n. Subsequently, these parameters were applied to the complete dataset. The default -m 3 was used as the minimum number of identical raw reads required to create a stack [53]. Mean coverage values were tracked within the de novo assembly pipeline at the end of the ustacks step, which takes the short-read sequences and aligns them into stacks, and the gstacks step, which identifies and genotypes and the SNPs within the metapopulation for each locus. Using the population program with a population map consisting of three groups (*S. alba*, *S. alba × babylonica*, and *S. babylonica*), the -r 80 parameter was applied as the minimum percentage of individuals in each population required to process a locus for that population. The -p 2 parameter was used as the minimum number of populations in which a locus must be present to be processed, considering that loci could be present in both the parental *S. alba* and hybrids, as well as in the parental *S. babylonica* and hybrids. The minimum minor allele count (–min-mac) required to process an SNP was set to 3. The maximum observed heterozygosity (–max-obs-het) was set to 0.6. Finally, the flag –write-single-snp was used to remove linkage disequilibrium by restricting data analysis to only the first SNP per locus. The resulting dataset will hereafter be referred to as the hybrid dataset. In addition, a second de novo pipeline, following the same parameters, was performed to include three samples of *S. triandra* as an outgroup for network analysis. VCFtools v.0.1.16 [54] was applied to both datasets using the –max-missing 0.95 flag to exclude sites below this threshold.

### 2.3. Analysis of Hybrid Structure

**Figure 2:**
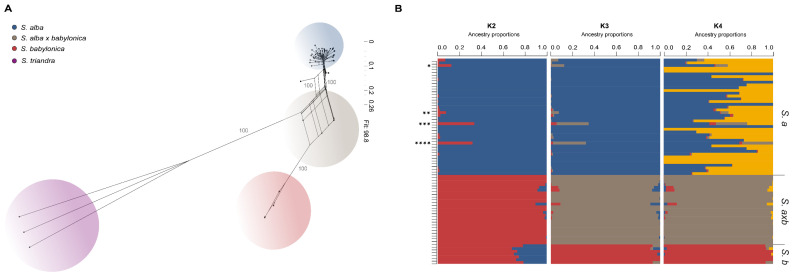
(**A**). Neighbor-Net network based on 14,294 unlinked SNPs from 77 *Salix* (=*S.*) samples (Fit: 98.8): *S. alba* (41), *S. alba × babylonica* (25), *S. babylonica* (7), and *S. triandra* (3) as an outgroup. Individuals are color-coded by population. Bootstrap values are shown on the connecting branches. The scale for branch lengths is indicated in the top right corner. (**B**). sNMF barplot based on 37,613 unlinked SNPs from 74 samples, including only the parental species and their hybrids. Ancestry proportions (Q-matrix) are shown for K = 2, 3, and 4. Species clusters are labeled on the right side of the plot: *S.a* for *S. alba*, *S. axb* for *S. alba × babylonica*, and *S.b* for *S. babylonica*. Four introgressed individuals are apparent in the K = 2 and 3 plots (*: EH 11235, **: EH 11261, ***: EH 11271, ****: EH 11379).

Using the dataset that included the *S. triandra* outgroup, a splits graph was reconstructed with SplitsTree App v.6.4.14 [55] using default parameters, including P-distance and Neighbor-Net (NN) analysis [56], with 1000 bootstrap replicates for statistical support (Figure 2A).

An sNMF analysis [57] was performed using the hybrid dataset to explore the presence of distinct genotype groups within the data, using the R packages and LEA v3.20.0 [58]. This method applies non-negative matrix factorization and least-squares optimization to estimate the number of ancestral populations (K). Ten different K values were tested, each with 1000 repetitions, with the ploidy parameter explicitly set to tetraploid (4n). The R package vcfR v1.15.0 [59] was used to convert the final dataset into formats compatible for downstream analyses. The optimal value of K was estimated using the cross-entropy criterion and further supported by a principal component analysis (PCA), performed with the R packages vcfR v.1.15.0 and dartR v2.9.9.5 [60] and visualized with ggplot2 v3.5.2 [61] (Supplementary Figure S1). The graphs for K2, K3 and K4 are shown in Figure 2B. 

**Figure 3:**
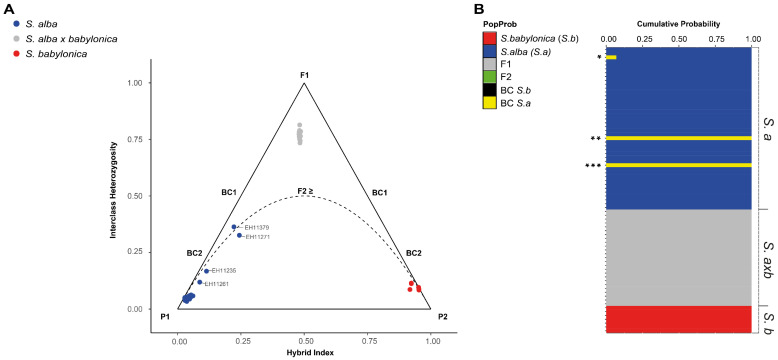
(**A**). Triangle plot generated from triangulaR analysis based on 37,613 SNPs on *Salix* (=*S.*). The Y-axis represents the proportion of interclass heterozygosity, while the X-axis shows the hybrid index. Individuals are color-coded by population. The expected theoretical positions of genotype classes are indicated within the triangle. Four individuals from the parental population of *S. alba* appear closer to the theoretical positions of BC2 (from bottom to top: *S. alba* EH11261 and EH11235) and BC1 (from bottom to top: *S. alba* EH11271, and EH11379). (**B**). NewHybrids barplot based on the hybrid dataset (74 samples). Columns represent cumulative genotype probabilities, and rows correspond to individual samples. Group names are indicated on the left: *S.a* for *S. alba*, *S.axb* for *S. alba × babylonica*, and *S.b* for *S. babylonica*. The color legend identifies two parental categories: Pure 1, which includes all *S. babylonica* individuals, and Pure 2, which includes most *S. alba* individuals. Individual EH11235 (*) carries only a minor genetic contribution from *S. alba*. Two individuals within the *S. alba* group (**: EH11271, and ***: EH11379) are highly supported as backcross 2 category (BC2). All other *S. alba × babylonica* hybrids are classified as first filial generation (F1) hybrids.

A triangle plot was generated using the hybrid dataset and the R package triangulaR v0.0.1 [62] (Figure 3A). This package includes functions to estimate interclass heterozygosity and the hybrid index of individuals to infer genotype categories (e.g., parental, F1, F2, backcrosses). *S. alba* and *S. babylonica* were defined as the parental species, and an allele frequency difference threshold of 0.5 between them was applied. After filtering, 17,446 variant sites remained and were used to calculate the hybrid index and interclass heterozygosity for each sample.

A complementary analysis on hybrid classes was performed using NewHybrids [63], applying 100,000 MCMC iterations with a burn-in period of 25,000. Due to computational constraints, this analysis was conducted on a subset of 300 randomly selected loci from the hybrid dataset (Figure 3B). The R package hybriddetective v0.1.0.9 [64] was used to plot the NewHybrids result.

### 2.4. Identification of Sex-Specific Genomic Sequences

An analysis to identify sex-specific genomic sequences and infer the sex determination system (SDS) of the hybrid population was performed using the hybrid dataset (see below) with the software RADSex v1.2.0 [43] and the R package sgtr v1.1.2 [65]. RADSex enables comparisons of RAD sequences between phenotypically distinct females and males, and more generally, between two groups, by performing presence/absence tests [65].

**Figure 4:**
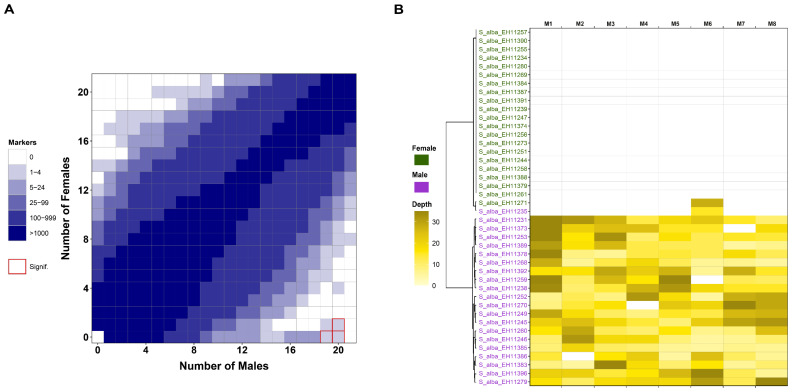
RADSex analyses on *Salix* (=*S.*) samples. (**A**). Tile plot resulting from the distrib RADsex analysis in *S. alba* samples. The distribution of RADSex markers between phenotypic females (Y-axis) and phenotypic males (X-axis) is shown. Tile color intensity indicates the number of markers present in the corresponding number of males and females. Tiles that exhibit a statistically significant association with phenotypic sex, based on a Chi-squared test (*p* < 0.05, Bonferroni correction), are indicated with a red border. The significant markers suggest a XX/XY male heterogametic sex determination system (SDS). (**B**). Heatmap resulting from the signif RADSex analysis in *S. alba* samples. Individuals (rows) are color-coded to distinguish phenotypic females from males. Each column corresponds to a different marker. Marker FASTA sequences can be found in Supplementary Figure S3. Depth is represented by a color gradient ranging from 0 (white, indicating absence) to ≥30 (olive yellow), with increasing depth shown by progressively more intense and darker shades. A rectangle on the left side of individuals’ ID is color-coded by population. Depth is represented by a color gradient ranging from white (indicating absence) to olive yellow, with increasing depth shown by progressively more intense and darker shades.

**Figure 5:**
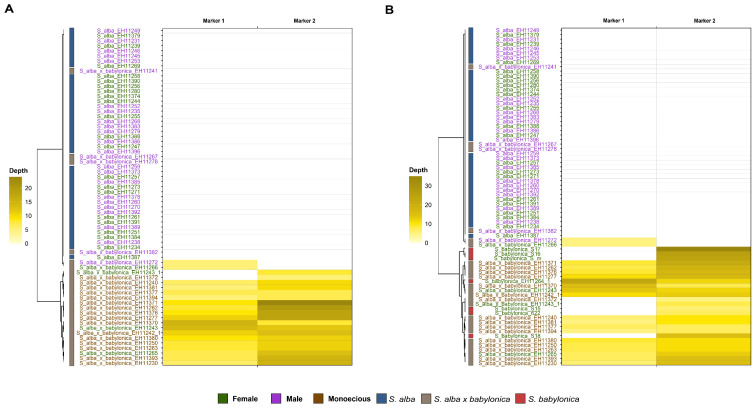
(**A**). Heatmap resulting from the exploratory signif RADSex analysis in *Salix* (=*S.*), *S. alba* + *S. alba* × *babylonica* samples. Individuals (rows) are color-coded to distinguish phenotypic females from males and monoecious hybrids. Each column corresponds to a different marker. (**B**). Heatmap resulting from the exploratory signif RADSex analysis in *S. alba* + *S.alba* × *babylonica* + *babylonica* samples. Individuals (rows) are color-coded to distinguish phenotypic females from males and monoecious hybrids. Each column corresponds to a different marker. Marker 1 and Marker 2 FASTA sequences can be found in Supplementary Figure S4 and Figure S5, respectively. Color coding as in Figure 4.

For this analysis, previously demultiplexed reads obtained using the process_radtags program [50] were used directly in the RADSex pipeline. The first step, using the process command, generated a table with sequence depth values for each marker in each individual. Three group comparisons were objectively exploratory analyzed based on several preliminary tests: (1) *S. alba* population (42 samples), comparison of females and males (Figure 4); (2) *S. alba* + *S. alba* × *babylonica* population (67 samples), comparison of males and monoecious individuals (i.e., individuals expressing both male and female phenotypes in the hybrid population) (Figure 5A); (3) complete hybrid dataset: *S. alba* + *S. alba* × *babylonica* + *S. babylonica* (total of 74 samples), comparing phenotypic males and monoecious individuals (Figure 5B). It is important to note that samples not directly involved in a given comparison (e.g., *S. babylonica*, composed entirely of females) were still included in the analysis; RADSex estimates for these samples were calculated and plotted alongside the group they most closely resembled [43].

A comparison within the *S. babylonica* population alone (7 samples) was not possible, as all individuals were female, and a second group is required for RADsex comparisons. Fortunately, the SDS of *S. babylonica* is already known [20]. Additional comparisons are summarized in Supplementary Table S2. Therefore, RADsex analysis was not feasible for those cases.

In the *S. alba* population, the distrib command was used to compute the distribution of markers between females and males (Figure 4A), and the signif command identified markers significantly associated with sex (Figure 4B). Both analyses were visualized using the sgtr R package [43]. In the *S. alba* + *S. alba* × *babylonica* and complete hybrid populations, the signif command was again used to detect sex-associated markers (Figure 5A and Figure 5B, respectively).

Finally, the FASTA output from the signif analysis was used in a web BLAST nucleotide search in NCBI to validate the identified sex-specific genomic sequences by comparison with publicly available *Salix* reference sequences (database: Nucleotide collection (nr/nt), organism (*Salix* taxid:40685) (Supplementary Figures S4 and S5).

## 3. Results

### 3.1. RAD-Loci Assembly Results

An average of 152,608 RAD loci and 106,511 variant sites, with a mean coverage of 25 × (after ustacks step) and 32.2 × (after gstacks step), were generated from the hybrid dataset using the de novo assembly pipeline. Filtering with VCFtools (–max-missing 0.95) reduced the hybrid dataset to 37,613 high-quality variant sites with a mean missing data of 1.38%. The second de novo assembly including three *S. triandra* samples as an outgroup yielded 152,614 loci and 106,489 variant sites. After applying the same VCFtools threshold, 14,294 variant sites were retained.

### 3.2. Analyses of Hybrid Origin

The Neighbor-Net network, based on 14,294 unlinked SNPs from 77 *Salix* samples, revealed clear genetic differentiation among the three main groups: (*S. alba*), *S. babylonica*, and their hybrids (*S. alba* × *babylonica*), with *S. triandra* forming a distinct outgroup (Figure 2A). Individuals are clustered by taxon, with hybrid samples forming an intermediate reticulated group between the two parental species. Bootstrap support for the major splits was high (100), and the network fit was 98.8%, indicating strong support for the inferred genetic relationships.

Population structure analysis using sNMF (Figure 2B) identified K = 3 as the best-supported model, based on the cross-entropy criterion and confirmed by the principal component analysis (PCA) (Supplementary Figure S2). This configuration clearly separated the three groups: one cluster each for *S. alba*, the hybrids, and the *S. babylonica* samples. Notably, four samples within the *S. alba* population exhibited different ancestry proportions compared to the rest of the group. At K = 2, samples were divided into three apparent clusters: one corresponding to *S. alba*, one to the hybrids, and a third group within *S. babylonica* showing mixed ancestry between *S. alba* and the hybrids. The expected 50:50 ancestry proportions in the hybrids were not observed. At K = 4, additional substructure was observed within the *S. alba* population, suggesting varying degrees of admixture or underlying population structure within that species.

The triangle plot revealed a clear separation between the selected parentals *S. alba*, *S. babylonica*, and their hybrids based on hybrid index and interclass heterozygosity (Figure 3). The *S. alba* × *babylonica* hybrid individuals were distributed between the two parental groups, at almost the top of the triangle, consistent with intermediate genetic composition of an early first filial generation (F1). Conversely, four individuals from the *S. alba* population (EH11261, EH11235, EH11271, and EH11379) showed unexpected patterns, clustering closer to the theoretical positions of backcross categories (BC1 and BC2). This pattern probably suggests past introgression affecting some *S. alba* individuals, or alternatively, these samples may represent outliers within the population. To further validate these findings, NewHybrids analysis on a random subset of 300 loci also supported that *S. alba* × *babylonica* individuals are F1 hybrids and the potential backcrosses or outliers EH11271, EH11379, and with only low support, also EH11235 (Figure 3B).

### 3.3. Sex Determination System (SDS) Analysis

The phenotypic identification revealed all *S. alba* trees as dioecious, with each of the 21 plants being male or female; the seven *S. babylonica* trees were all female. The hybrid *S. alba* × *babylonica* comprised 4 female, 6 male, and 15 monoecious trees, the latter with separate female and male catkins, or mixed catkins with female and male flowers (Figure 1); we did not observe hermaphroditic flowers. Stamens and pistils were normally developed, and female fruiting catkins regularly produced seeds.

In *S. alba*, the distribution of RADSex markers differed between phenotypic females and males, with significant markers detected in male individuals (Figure 4A). Eight markers showing a statistically significant association with sex (Figure 4B). These findings support a male heterogametic (XX/XY) sex determination system in this species. Marker depth patterns clearly distinguished males from females, highlighting sex-specific genomic regions.

In the *S. alba* + *S. alba* × *babylonica* dataset, the exploratory analysis using RADSex identified two markers with depth patterns clearly associated with phenotypic sex (Figure 5A). These sex-specific markers showed differences in presence/absence and read depth between males, females, and monoecious individuals. The phenotypically male individuals from the hybrid population clustered with the *S. alba* population, while the phenotypically female hybrids clustered with the monoecious hybrid group sex (Figure 5A). When the analysis was extended to include the full hybrid dataset—*S. alba*, *S. alba* × *babylonica*, and *S. babylonica*—the same two sex-specific markers remained consistently associated with sex phenotype across populations (Figure 5B). These results provide some evidence for a combined sex determination system in the hybrid group *S. alba* × *babylonica*, with phenotypically male hybrids clustering with *S. alba*, and phenotypically female hybrids aligning more closely with the monoecious hybrid group, which also includes the female *S. babylonica* samples. The FASTA sequences corresponding to sex-specific markers in Figure 5 are available in Supplementary Figures S4 and S5. Hybrids *S. alba* × *babylonica* present both Marker 1 and Marker 2. Pure *S. babylonica* samples present only Marker 2 (Figure 5B). The BLAST analysis of Markers 1 and 2 revealed 100% identity and low e-values when aligned to the reference *S. babylonica* genome. Marker 1 matched autosome 19, while Marker 2 aligned with the sex chromosome 15, both reported in [20].

## 4. Discussion

This study aimed to elucidate the hybrid status and reproductive characteristics of one of the historically cultivated *Salix* hybrids in Central Europe, the weeping willows (*S. alba* × *babylonica*) [66]. Since Mendel’s early research on hybrids in plants [27,31,67,68], recent studies of the *Salix sericea* Marsh.–*Salix eriocephala* Michx. hybrid complex [27,31,67,68], studies of the polyploid hybrid complex *Salix alba* L.-*Salix fragilis* L. [27,31,67,68], and investigations of hybrids between *S. foetida* and *S. waldsteiniana*, and *S. alpina* and *S. breviserrata* [27,31,67,68], have shown that hybrids often do not represent the precise intermediate phenotypes and genotypes between the parental species [27,31,67,68]. It is also known that hybrid populations exhibit a spectrum of genotypes, which can lead to misinterpretation when individuals are classified into discrete genetic categories without carefully considering whether such classification is biologically realistic [69]. To address this, we investigated the hybrid category of weeping willows using RADseq within a de novo methodology. Despite Stacks identifying only biallelic SNPs within each locus, and the studied samples being tetraploid, we optimized parameters for loci assembly to maximize the number of informative polymorphic loci, and we were able to achieve an accurate classification of hybrid status. The final mean coverage value (32.2×) supports the reliable detection of different alleles at a specific locus even for tetraploids [70,71]. Furthermore, by selecting only one SNP per locus, we eliminate the bias introduced by merging different polyploid subgenomes in the analyses used to infer the hybrid category of our samples [43]. In parallel, we examined the reproductive systems of these hybrids, with particular focus on the occurrence of monoecy, a deviation from the dioecy that predominantly characterizes *Salix* species. Notably, the use of RADSex enabled us to move beyond traditional genetic mapping, which is commonly used to identify sex-specific genomic sequences, by instead detecting these sequences through direct comparisons between phenotypic males, females, and monoecious plants. We find support for the hypothesis that the hybrid combined a ZW system inherited from *S. babylonica* and a XY system inherited from *S. alba*.

### 4.1. Genetic Composition of Hybrids and Comparison of Different Methods

In the Neighbor-Net analysis (Figure 2A), the *Salix alba* × *babylonica* artificial hybrid samples are represented entirely by a reticulated network [55,72]. This pattern suggests recent hybridization events, consistent with historical expectations [11]. Unlike the pure parental populations (*S. alba* and *S. babylonica*), and unlike natural or long-term hybrids that have progressed toward speciation (e.g., allopolyploid *S. caesia* positioned between *S. purpurea* and *S. repens* in the Neighbor-Net [70]), the *S. alba* × *babylonica* hybrids do not form extended or pronounced branches in the network.

While the Neighbor-Net supports a hybrid origin for *S. alba* × *babylonica*, results from sNMF (Figure 2B) and PCA analyses (Supplementary Figure S2) suggest that these individuals are not typical F1 hybrids, which would be expected to show ~50:50 ancestry proportions from each parental species (Figure 2B, K = 2). Instead, at K = 3 and K = 4 (Figure 2B), *S. alba* × *babylonica* appears as a distinct lineage. A similar result of *S. alba* × *babylonica* representing a separate genetic cluster was found in Czech Republic [29], based on STRUCTURE analysis and DArTSeq markers. These findings suggest that clustering methods such as sNMF or STRUCTURE, even when complemented with PCA, may be limited in their ability to infer hybrid categories from the allele-based ancestry matrix [62,63,69,73,74,75], at least in artificial early-generation homoploid hybrids.

In the sNMF results (Figure 2B), most hybrid individuals are treated as a new discrete genetic cluster (K = 3 and 4). The resulting barplots do not reflect proportional contributions from the parental species, nor do they allow clear genotype classification into categories such as parentals, F1, F2, or backcrosses [62,69,74,75]. Focusing on the *S. alba* cluster (Figure 2B, K = 2 and 3), the bar plot suggests the presence of four introgressed samples (*: *Salix alba* EH11235; **: EH11261; ***: EH11271; ****: EH113179). However, combined evidence from subsequent genetic analyses and morphological observations made during sampling is needed for a correct genotype classification of these individuals (see below). Interestingly, the ancestry matrix at K = 2 (Figure 2B) reveals a pattern within the *S. babylonica* group (7 samples) that echoes prior findings [75]: “in groups that contain fewer samples (…) if an ancient sample is included among modern individuals, it is typically represented as an admixture of the modern populations”.

Furthermore, comparing the topologies observed in the Neighbor-Net—with *S. babylonica* forming a compact cluster, *S. alba* × *babylonica* showing reticulation, and *S. alba* displaying broader dispersion (Figure 2A)—to the increasing K values in the sNMF analysis (Figure 2B and Supplementary Figure S2), it becomes apparent that increasing K does not resolve the ancestry proportions of hybrid samples. Instead, it seems to primarily reveal intraspecific genetic variation, particularly within *S. alba*, highlighting a previously noted issue with applying interspecific methods at the intraspecific level (“tokogeny” rather than a phylogeny model) [72]. This correspondence between Neighbor-Net topology and population-level structure shown in bar plots has also been evaluated in [76] and seen in other *Salix* studies, for example in *S. foetida* [30].

A critical issue lies not only in the allele-based clustering methodology but also in the prior assumptions of researchers, who often expect discrete genotype classifications. This overlooks the reality that hybrids frequently exist along a continuum or cline, rather than as clearly separable categories [69]. Nonetheless, the sNMF analysis at K = 3 (Figure 2B) and the PCA (Supplementary Figure S1) suggest the presence of a genetically distinct cluster, as is previously found in homoploid hybrids [77]. From this perspective, we propose that this is a case where K-model-based clustering methods (e.g., sNMF or STRUCTURE) may tend to classify these types of hybrids into two broad categories: “pure” vs. “hybrid” [69]. We therefore caution against using only K-model clustering, PCA, and Neighbor-Net analyses in isolation for genotype classification of hybrids [75]. These tools are valuable for understanding overall genetic relationships but are not designed to determine specific hybrid classes (e.g., F1, F2, backcrosses). Finally, considering that sNMF reflects results at an allele level, we suggest that the hybrid cluster revealed by sNMF is likely the result of mutations occurring shortly after the hybridization event. However, this hypothesis requires further analysis using methods that infer SNP categories (e.g., SNiploid) [70] or different sequencing approaches beyond reduced representation sequencing (RRS). This phenomenon is plausible in polyploids and has been previously documented in both natural and artificial populations [78].

The triangulaR approach, when applied to RADseq data, allows the analysis not only of alleles (17,446 SNPs, using an allele frequency difference threshold of 0.5) but also the proportion of loci inherited from each parental, and remains effective even with small sample sizes, as few as five individuals, without compromising accuracy [62], which is relevant in our study given that the *S. babylonica* population includes only seven samples. Sample size per parental group is crucial, as noted in [62]. However, they also reported that the optimal number depends on factors like the genetic divergence of parental species. Diverged species, such as *S. babylonica* and *S. alba*, require fewer samples. Although both species are in the same subgenus *Salix* s.l. [5], they have distinct evolutionary histories [5,34,35]. The triangle plot incorporates two key metrics: the interclass heterozygosity (the proportion of loci carrying alleles from both parental populations) and the hybrid index (the proportion of alleles matching either *S. alba* or *S. babylonica* parental frequencies) [69]. We found the inclusion of the proportion of loci inherited from the parental species to be valuable, especially when working with tetraploids. Due to the diploid base calling and phasing approach in Stacks, only the two most plausible alleles out of four are selected. By using triangulaR, we can infer hybrid categories not only at the allele level but also at the locus level. Furthermore, the pattern observed in our artificial hybrids closely resembles the scenario simulated in [74], which modeled a population without introgression.

Our triangle plot result suggests that the artificial *S. alba* × *babylonica* hybrids do not extend beyond the first hybrid generation (F1), even when a relaxed allele frequency difference threshold of 0.5 is applied [74]. This interpretation fits the expectation from the cultivation history, with first artificial crosses made in the late 19th century [11], and only planted occurrences in Central Europe without any trees that escaped into natural habitats. The F1 hybrid status is further supported by the Bayesian-based NewHybrids analysis [63], a commonly adopted strategy for distinguishing hybrid classes (Figure 3B). NewHybrids accounts for genotype frequencies in its clustering approach, offering reliable classification especially in the first hybrid generations [7,28], but at a high computational cost when dealing with the large numbers of loci typical of RADseq datasets [63].

Interestingly, the triangle plot and the NewHybrids analysis identified two individuals as backcrosses to *S. alba*. EH11271 showed a typical *S. alba* phenotype, except for the unusual low number of ovules (Appendix A), which is regarded as a good diagnostic character for hybrid identification [45,46]. This phenotypically female tree grows in the vicinity of a male hybrid tree and also had one male-specific SDS marker (see below). EH11379, a young female tree, had apically pendulous branches, and occurred at a roadside, only a few hundred meters distance from pure *S. babylonica* (EH11264) and a big old monoecious hybrid tree, making introgression plausible (Figure S1). We confirm the rare cultivation of *S. babylonica* in Central Europe [29]; *S. alba* individuals introgressed by *S. babylonica* were also reported in Czech Republic according to STRUCTURE analyses [29]. Other outliers in molecular analyses (EH11235 and EH11261) were not confirmed by NewHybrids. They had *S. alba* phenotypes, with some characters matching “*S. excelsa* C.C. Gmel.”, a taxon native to Central Asia that is probably just a genetic variant of *S. alba* [25,29]. These tall, slender trees (up to 30 m; Figure 1i) are sometimes cultivated in C. Europe and hybridize with *S. alba* [25]. From a bioinformatic perspective, these *S. alba* samples may represent cases where allelic dropout affected genotype inference [79] as suggested by their dispersion in the PCA (Supplementary Figure S2), the outlier proportion in the sNMF, and in the triangle plot analysis (Figure 2B and Figure 3A and Supplementary Figure S3). Also, the primarily diploid design of the Stacks pipeline, combined with the polyploid nature of the analyzed species, may contribute to the suggested allelic dropout. Furthermore, the intraspecific variation within *S. alba* seems to result in overlapping genetic signals with hybrid classes, complicating the distinction between backcrosses and purebreds. Consequently, only the two *S. alba* individuals that appear near the theoretical backcross 1 region in the triangle plot—also identified as outliers in the sNMF, PCA, and confirmed as backcross in NewHybrids analyses—may represent actually introgressed individuals.

### 4.2. Putative Sex Determination System (SDS) in S. alba and the Combined SDS in S. alba × S. babylonica Hybrids

Figure 4A provides clear evidence that *S. alba* likely follows a male heterogametic sex determination system (XX/XY or XX/X0), supported by the identification of eight sex-linked markers (Figure 4B, M1–M8) [43]. Male heterogametic SDSs (XY on chromosome 7) were also observed in *S. dunnii*, another species of *S.* subg. *Salix* [19]. Only two outliers were observed: one phenotypically female individual (*S. alba* EH11271) that shares a male-associated locus and is probably introgressed, and one phenotypically male individual (EH11235) that clusters within the female group in the dendrogram and which we regard an outlier (see above) (Figure 4B).

Figure 5 presents an exploratory analysis comparing two different sex determination systems (SDSs) using the RADSex approach. Because it allows statistical comparison of datasets using straightforward presence/absence tests, we suggest that this method seems capable of identifying sex-specific genomic sequences, including those unique to the *S. alba* sex determination system and those shared with hybrids and *S. babylonica*. In this context, it is possible to investigate the hybrid SDS by comparing genomic sequences from phenotypically confirmed males—likely reflecting the proposed male heterogametic system of *S. alba* (Figure 4)—and monoecious hybrids that likely carry components of both SDS, including that of *S. babylonica*, which is known to follow a female heterogametic system (ZZ/ZW) [20]. Interestingly, phenotypically confirmed hybrid males cluster with *S. alba* males in both Figure 5A,B, while phenotypically confirmed hybrid females cluster with *S. babylonica* females in Figure 5B. This suggests that the hybrid individuals may have inherited different sex-linked regions (SLRs) from both parental species.

**Figure 6:**
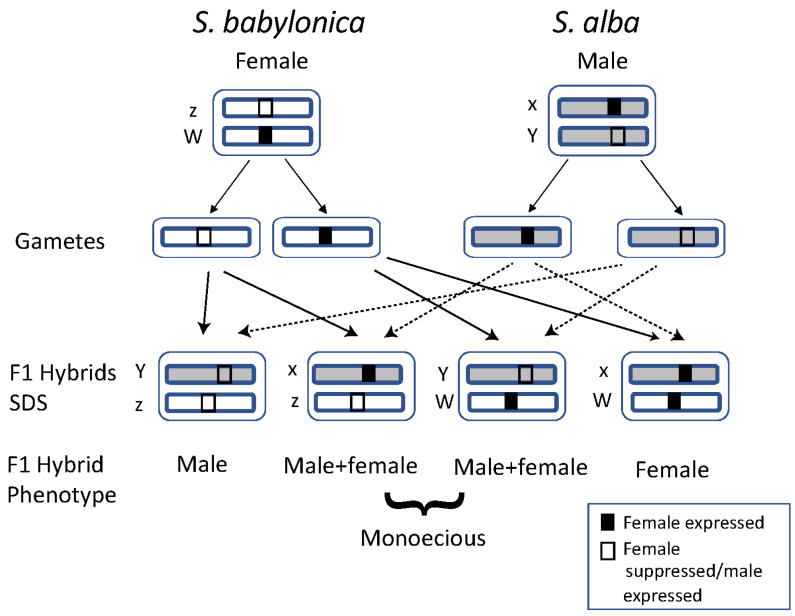
Hypothetical scheme of the SDS in the *S. alba* × *babylonica* hybrids, with the assumption of a single sex chromosome pair in tetraploids [23]; the other chromosome copies that have reverted to autosomes are not shown. In willows, genetic control of sexes is based on a single-locus system with a dominant W-linked factor for femaleness in ZW systems and a dominant Y-linked factor suppressing femaleness in XY systems [19,20,80], symbolized by black and white boxes, respectively. From the gamete combinations, four SDS configurations are possible in the F1 hybrid: Yz is expected to be a male phenotype due to the Y system, xW female with the W acting, while xz and YW genotypes should express both sexes, resulting in monoecy. Arrows with dotted lines used for gametes from male parent.

The concordance between phenotypic and now molecularly inferred “pure” male and female *S. alba × babylonica* hybrids, and monoecious plants, may reflect different chromosomal configurations in the hybrid offspring. We assume a single sex chromosome copy in tetraploids in the heterogametic sex as typical for polyploids [23], and a dominant single-locus system in willows [19,20,80]. The female parent *S. babylonica*, which was used for crossings in Central Europe [32], would have zW, the male *S. alba* xY sex chromosomes (Figure 6). From the four possible offspring classes, two would be probably monoecious, one female, and one male; see Figure 6. Strikingly, the observed proportions of sex phenotypes in the hybrids, with a majority of monoecious plants, roughly fits this expectation. Controlled crosses and offspring analyses, more detailed investigations of the sex-linked region-bearing chromosomes, and gene expression analyses in the hybrids will be needed to confirm this hypothesis. On the other hand, the phenotype might be influenced also by environmental sex determination (ESD) [16], which could explain proterandry in the monoecious trees. Other effects are unlikely, as the sampling area is quite uniform regarding climatic and topographic conditions, and all hybrid plants were in cultivation.

Moreover, the two markers that show significant association with sex in both Figure 5A,B is the same and particularly informative. Most hybrid individuals carry both markers, whereas *S. babylonica* females tend to carry only one of them at high sequencing depth (e.g., *S_babylonica_S17*, *S_16*, and *S_m*). BLAST complementary analysis revealed that Marker 2 is located on a known sex chromosome (chromosome 15), while Marker 1 aligns with a somatic chromosome (chromosome 19) in *S. babylonica* [20] (Supplementary Figures S4 and S5). These molecular findings align with known patterns in *S. alba × babylonica*, a hybrid here documented with diversity in sexual expression, including males, females, and monoecious individuals. We propose that the patterns observed in Figure 5 represent a first glimpse into the SDS of this artificial hybrid population, which appears to result from the combination of two distinct parental SDS. This scenario mirrors historical transitions in sex chromosome evolution, as suggested for allotetraploid *S. babylonica* in previous studies [20]. In *Mercuralis perennis*, a shift from dioecy to monoecy occurred during allopolyploidization [22]. So far, the breakdown of dioecy and a shift to monoecy in one- or two-locus systems was usually ascribed to mutations or losses of sex-determining genes [16]. Here we show for the first time that hybridization of different SDSs can result in monoecy already in the primary hybrids, which may contribute to our understanding of the evolution of SDSs.

## 5. Conclusions

Based on our data, we reaffirm the classification of our *S. alba* and *S. babylonica* samples as genetically pure parentals and the *S. alba* × *babylonica* individuals as F1 homoploid hybrids. We demonstrate that RADseq, using a de novo pipeline with optimized parameters and sufficient coverage, effectively resolves hybrid categories in tetraploid homoploid artificial hybrids. Our findings also highlight the increasing availability of tools for hybrid classification. Future studies should select analytical methods based on their suitability for the specific research questions being addressed.

These hybrids also represent an early shift toward monoecy, a condition potentially derived from the dioecious ancestral condition of the genus and both parental species [20]. This shift likely originated from the combination of an XY system from *S. alba* and ZW system from *S. babylonica*, as revealed through comparisons between phenotypic males, females, and monoecious individuals. We used the RADSex tool and found it effective for identifying sex-specific genomic sequences. Our results offer preliminary insights into putative sex-linked genomic regions in the artificial hybrid population (Figure 5, Supplementary Figures S4 and S5), and the evolution of SDSs in early-generation hybrids.

## Data Availability

The raw sequencing data are available at NCBI GenBank SRA under Bioproject accession number PRJNA1283918.

## Supplementary Materials

The following supporting information can be downloaded at: https://www.mdpi.com/article/10.3390/genes16080958/s1, Figure S1: Maps of collection sites in Europe; Figure S2: sNMF K values and PCA analyses; Figure S3: sNMF barplot for K = 5–10; Figure S4: BLAST analysis of Marker 1 from Figure 5; Figure S5: BLAST analysis of Marker 2 from Figure 5. Table S1: Materials used in this study; Table S2: RADSex comparison summary.

## Abbreviations

The following abbreviations are used in this manuscript:

*S.*	*Salix*
SDS	sex determination system
RADSeq	Restriction-site Associated DNA Sequencing
DArTseq	Diversity Array Technology
NCBI	National Center for Biotechnology Information (https://www.ncbi.nlm.nih.gov/, accessed on 16 March 2025)

## Appendix A

**Table A1:** Diagnostic characters of *S. alba*, *S. babylonica* and the weeping willow *S. alba* × *babylonica* ^1^.

Character	*S. alba*	*S. babylonica*	*S. alba* × *babylonica*
Height	Up to 30 m	Up to 18 m	Up to 20 (30) m
Habit	Branchlets erect, sometimes apically slightly pendulous	Branchlets erect to pendulous ^2^	Branchlets long pendulous from the basis
Branchlets	Brown, appressed hairy	Brownish, glabrous	Yellow, glabrous
Leave shape	Lanceolate	Narrow lanceolate to linear lanceolate	Narrow lanceolate to linear lanceolate
Stipules	Caduceous	Present during anthesis, ovate-lanceolate	Mostly caduceous; rarely present at anthesis, ovate-lanceolate
Leave indumentum (lower surface)	Densely silky hairy	Glabrous or sparsely pilose	Sparsely pilose, glabrescent
Sexes	Dioecious	Dioecious	Monoecious or dioecious
Female catkins (after anthesis)	3.0–6.0 cm long	2.0–3.0 cm long	3.0–5.5 cm long
Ovary	Glabrous	Glabrous to hairy	Glabrous
Ovule no. per ovary	8–24 ^3^	2–6	2–8

^1^ Compiled after collected material and literature [11,32,33,36,45,46]; ^2^ The individual found in Germany (EH11264) was a cultivar with tortuose branchlets; ^3^ The introgressed plant EH11271 had just 4–6 ovules per ovary.
