# Supplementary material for: Hybridization Resulted in Shifts from Dioecy to Monoecy in Weeping Willows (Salix L.)
Source: Genes (Basel). 2025 Aug 13;16(8):958. doi: 10.3390/genes16080958 (PMC12385670; doi:10.3390/genes16080958)
Supplement: Supplementary file 1 [file genes-16-00958-s001.zip › genes-3747851-supplementary.pdf]

Supplementary Materials

The following supporting Information is available for this article:

Salix = *S.*

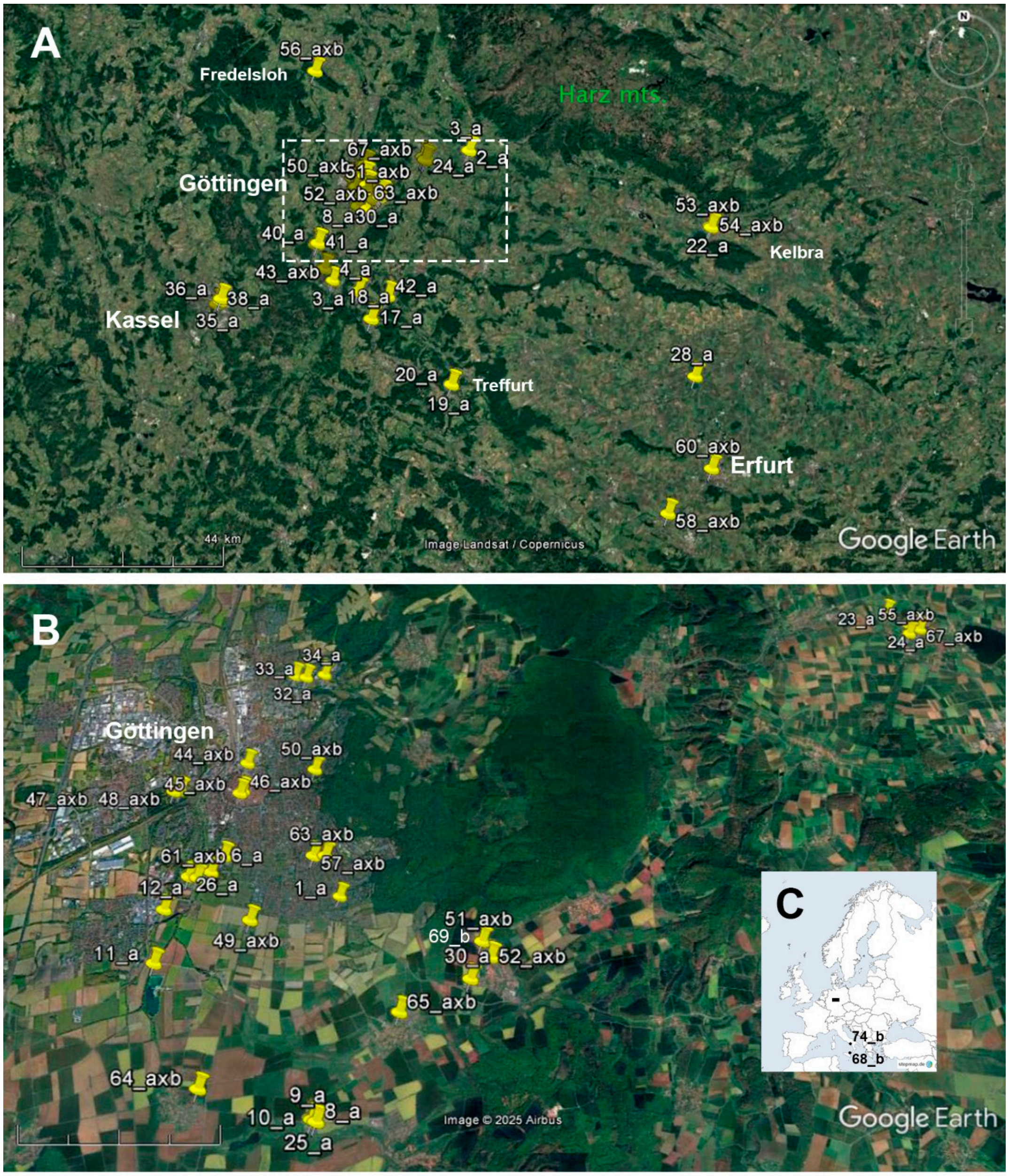

Figure S1. Maps of collection sites in Europe. A, Sampling area in Central Europe, with white rectangle indicating close-up of B; B, close-up of sampling area around Göttingen; C, Europe, showing location of Central European sampling area of A (black rectangle) and the two *S. babylonica* locations in Italy. Numbers correspond to running sample number in Appendix A; a = *S. alba*, b = *S. babylonica*, axb = *S. alba* × *babylonica*. Detailed coordinates and sexes are given in Table S1.

A

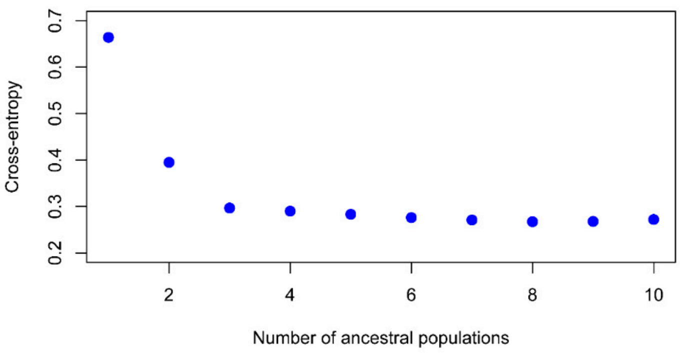

B

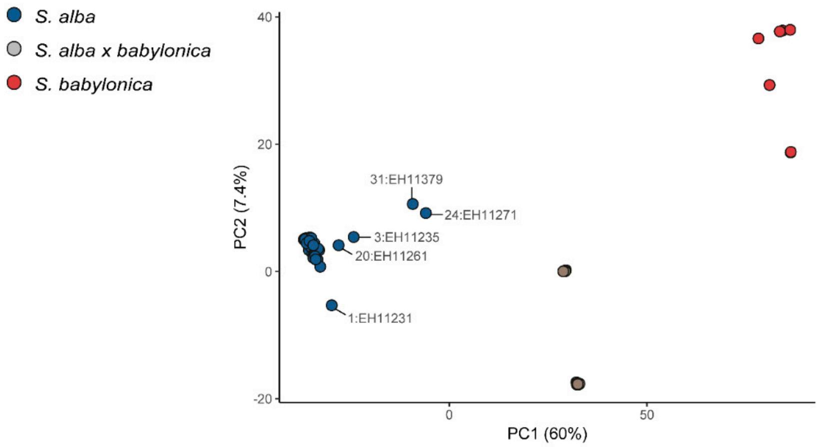

Figure S2. sNMF *K* values and PCA analyses. **A.** showing the cross-entropy values as a function of the number of ancestral populations (K) in the *sNMF* analysis of the hybrid dataset (74 samples), generated with the *LEA* R package. The optimal number of clusters is inferred to be K = 3, corresponding to the "knee" point of the curve. **B.** Principal component analysis (PCA) of the hybrid dataset showing three distinct clusters: one corresponding to the *S. alba* population, one to the *S. alba* × *babylonica* hybrids, and one to the *S. babylonica* population. A few *S. alba* individuals appear as outliers and are labeled for identification in the *sNMF*, *NewHybrids*, *triangulaR* plot, and *RADSex* analyses. The numbers to the left of each sample name (1:EH11231, 3:EH11235, 20:EH11261, 24:EH11271, and 31:EH11379) indicate their position in the *sNMF* barplot and *NewHybrids* output, facilitating cross-referencing across analyses.

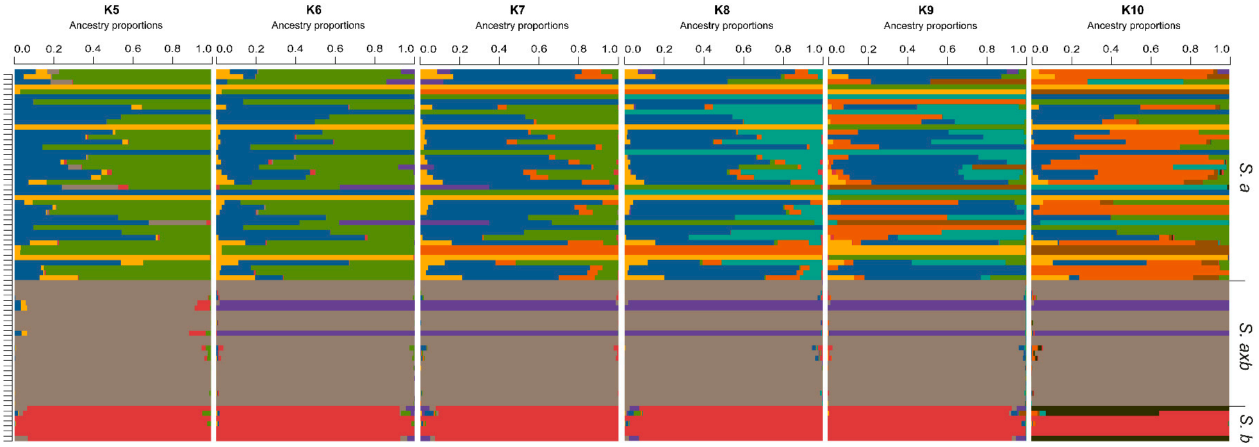

Figure S3. ***sNMF*** **barplot for** ***K*** **= 5-10**. Analyses based on 37,613 unlinked SNPs from the hybrid data set (74 samples), including only the parental species and their hybrids. Ancestry proportions (Q-matrix) are shown for *K* = 5 to 10. Columns within the subplots represent the range of ancestry proportions, and rows correspond to individual samples. Species clusters are labeled on the right side of the plot: *S.a* for *S. alba*, *S.axb* for *S. alba* × *babylonica*, and *S.b* for *S. babylonica*. Notably, the *S. alba* cluster displays a high degree of intraspecific variation.

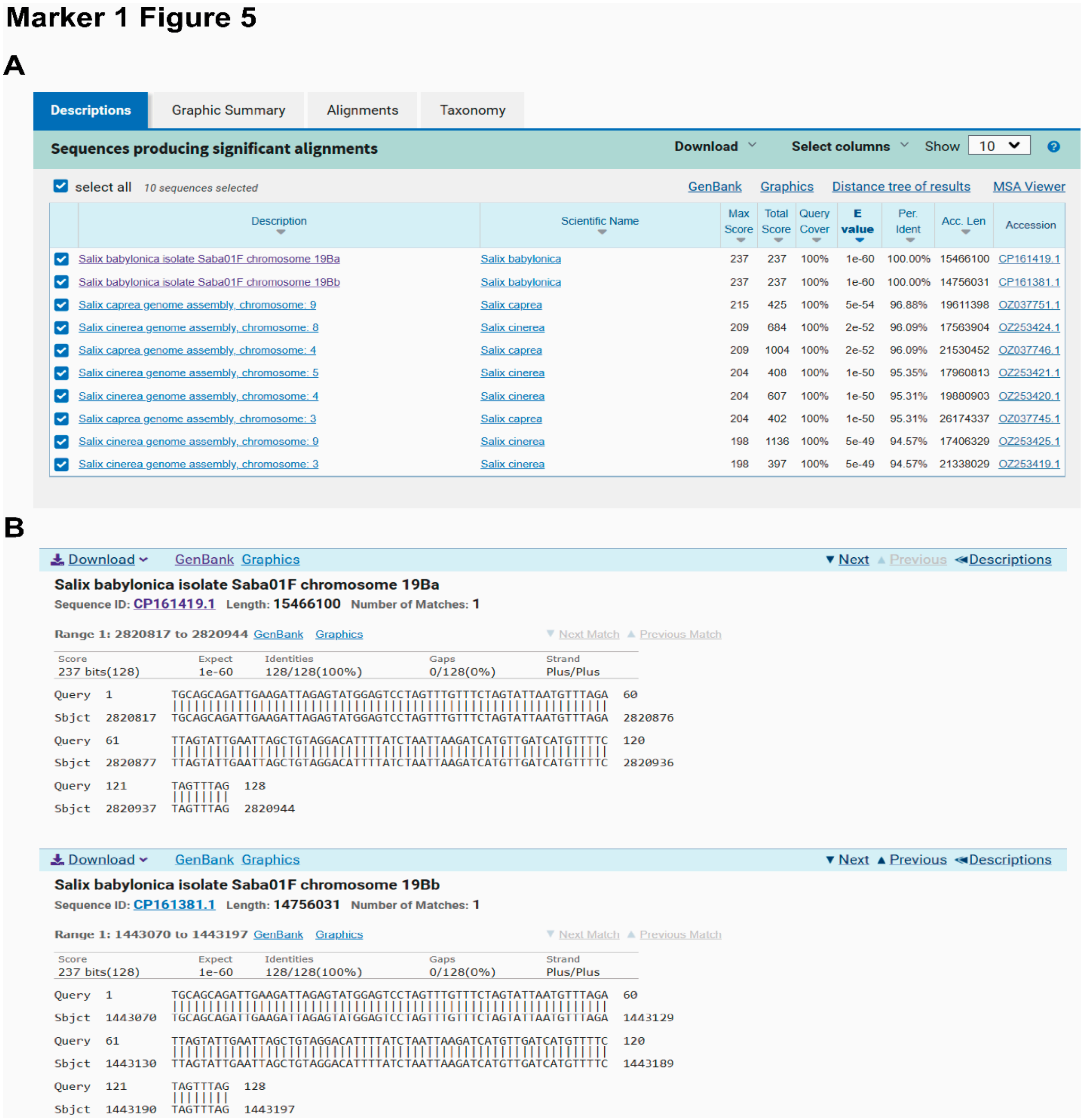

Figure S4. BLAST analysis of Marker 1 from Figure 5. A. First ten results from the BLAST search, showing the best e-values and percentage identity, with the highest-scoring matches corresponding to two accessions on autosome 19, published in (He, et al., 2024. 10.1038/s41467-024-51158-3). B. Alignment of Marker 1's sequence to the reference sequence on chromosome 19.

Marker 2 Figure 5

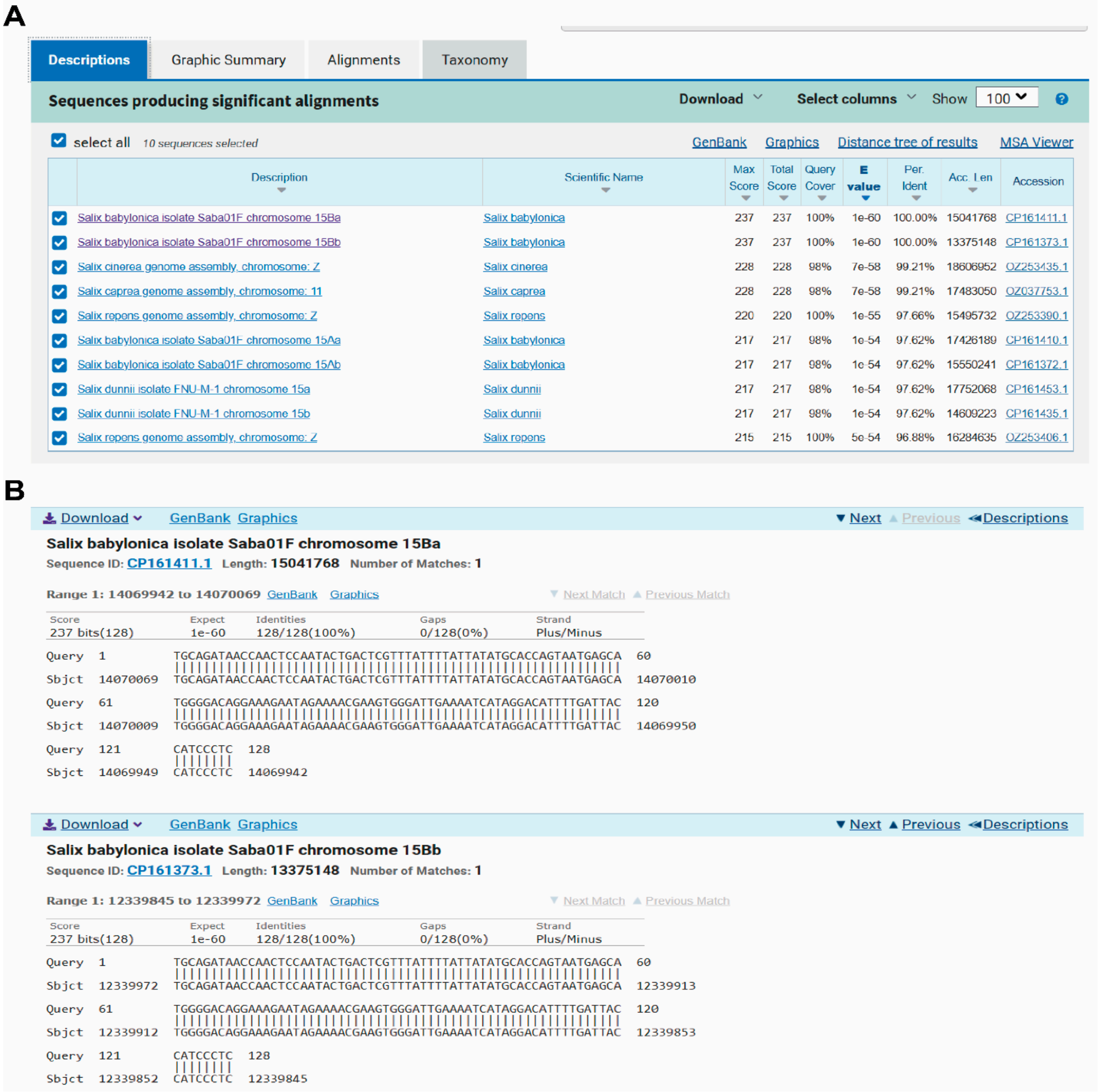

A

Descriptions | Graphic Summary | Alignments | Taxonomy

Sequences producing significant alignments — Download — Select columns — Show 100

select all *10 sequences selected* — GenBank — Graphics — Distance tree of results — MSA Viewer

	Description	Scientific Name	Max Score	Total Score	Query Cover	E value	Per. Ident	Acc. Len	Accession
☑	Salix babylonica isolate Saba01F chromosome 15Ba	Salix babylonica	237	237	100%	1e-60	100.00%	15041768	CP161411.1
☑	Salix babylonica isolate Saba01F chromosome 15Bb	Salix babylonica	237	237	100%	1e-60	100.00%	13375148	CP161373.1
☑	Salix cinerea genome assembly, chromosome: Z	Salix cinerea	228	228	98%	7e-58	99.21%	18606952	OZ253435.1
☑	Salix caprea genome assembly, chromosome: 11	Salix caprea	228	228	98%	7e-58	99.21%	17483050	OZ037753.1
☑	Salix repens genome assembly, chromosome: Z	Salix repens	220	220	100%	1e-55	97.66%	15495732	OZ253390.1
☑	Salix babylonica isolate Saba01F chromosome 15Aa	Salix babylonica	217	217	98%	1e-54	97.62%	17426189	CP161410.1
☑	Salix babylonica isolate Saba01F chromosome 15Ab	Salix babylonica	217	217	98%	1e-54	97.62%	15550241	CP161372.1
☑	Salix dunnii isolate FNU-M-1 chromosome 15a	Salix dunnii	217	217	98%	1e-54	97.62%	17752068	CP161453.1
☑	Salix dunnii isolate FNU-M-1 chromosome 15b	Salix dunnii	217	217	98%	1e-54	97.62%	14609223	CP161435.1
☑	Salix repens genome assembly, chromosome: Z	Salix repens	215	215	100%	5e-54	96.88%	16284635	OZ253406.1

B

Download — GenBank Graphics — ▼ Next ▲ Previous ◀◀ Descriptions

Salix babylonica isolate Saba01F chromosome 15Ba

Sequence ID: CP161411.1 Length: 15041768 Number of Matches: 1

Range 1: 14069942 to 14070069 GenBank Graphics — ▼ Next Match ▲ Previous Match

```
Score           Expect   Identities      Gaps       Strand
237 bits(128)   1e-60    128/128(100%)   0/128(0%)  Plus/Minus

Query  1         TGCAGATAACCAACTCCAATACTGACTCGTTTATTTTATTATATGCACCAGTAATGAGCA  60
                 ||||||||||||||||||||||||||||||||||||||||||||||||||||||||||||
Sbjct  14070069  TGCAGATAACCAACTCCAATACTGACTCGTTTATTTTATTATATGCACCAGTAATGAGCA  14070010

Query  61        TGGGGACAGGAAAGAATAGAAAACGAAGTGGGATTGAAAATCATAGGACATTTTGATTAC  120
                 ||||||||||||||||||||||||||||||||||||||||||||||||||||||||||||
Sbjct  14070009  TGGGGACAGGAAAGAATAGAAAACGAAGTGGGATTGAAAATCATAGGACATTTTGATTAC  14069950

Query  121       CATCCCTC  128
                 ||||||||
Sbjct  14069949  CATCCCTC  14069942
```

Download — GenBank Graphics — ▼ Next ▲ Previous ◀◀ Descriptions

Salix babylonica isolate Saba01F chromosome 15Bb

Sequence ID: CP161373.1 Length: 13375148 Number of Matches: 1

Range 1: 12339845 to 12339972 GenBank Graphics — ▼ Next Match ▲ Previous Match

```
Score           Expect   Identities      Gaps       Strand
237 bits(128)   1e-60    128/128(100%)   0/128(0%)  Plus/Minus

Query  1         TGCAGATAACCAACTCCAATACTGACTCGTTTATTTTATTATATGCACCAGTAATGAGCA  60
                 ||||||||||||||||||||||||||||||||||||||||||||||||||||||||||||
Sbjct  12339972  TGCAGATAACCAACTCCAATACTGACTCGTTTATTTTATTATATGCACCAGTAATGAGCA  12339913

Query  61        TGGGGACAGGAAAGAATAGAAAACGAAGTGGGATTGAAAATCATAGGACATTTTGATTAC  120
                 ||||||||||||||||||||||||||||||||||||||||||||||||||||||||||||
Sbjct  12339912  TGGGGACAGGAAAGAATAGAAAACGAAGTGGGATTGAAAATCATAGGACATTTTGATTAC  12339853

Query  121       CATCCCTC  128
                 ||||||||
Sbjct  12339852  CATCCCTC  12339845
```

Figure S5. BLAST analysis of Marker 2 from Figure 5. **A.** Top ten results from the BLAST search showing the best e-values and percentage identity, with the highest-scoring matches corresponding to two accessions on sexual chromosome 15, published in (He, et al., 2024. 10.1038/s41467-024-51158-3). **B.** Alignment of Marker 2's sequence to the reference sequence on chromosome 19.

Table S1. Materials used in this study. Nr corresponds to number in Figure S1; Taxon and sampling ID corresponds to herbarium voucher; Category after New Hybrids result (Figure 3B); Sex: m = male, f = female; Country: G = Germany, I = Italy, Ch = China; Altitude: meters above sea level.

Nr	Taxon and sampling ID	Category	Sex	Country	Locality	Altitude (m a.s.l)	Coordinates	Field Observation	Collection Date(s)	Collector(s)
1	S_alba_EH11231	Pure2	m	G	Göttingen, Geismar	209	51°30'31.97"N, 9°57'46.28"E	c. 10 m tree, apically pendulous;	29.04.2023/12.05.2025	E. Hörandl
2	S_alba_EH11234	Pure2	f	G	Rüdershausen	156	51°34'57.71"N, 10°16'29.45"E	c. 30 m tree, erect	30.04.2023	E. Hörandl & al.
3	S_alba_EH11235	Pure2	m	G	Rüdershausen	156	51°34'54.98"N, 10°16'30.32"E	c. 30 m tree, erect "excelsa"	30.04.2023	E. Hörandl & al.
4	S_alba_EH11238	Pure2	m	G	Oberrieden	140	51°18'44.3 N, 9°55'48.6"E	c. 20 m tree, erect	01.05.2023	E. Hörandl & al.
5	S_alba_EH11239	Pure2	f	G	Oberrieden	140	51°18'45.02"N, 9°55'53.70"E	c. 20 m tree, erect	01.05.2023	E. Hörandl & al.
6	S_alba_EH11244	Pure2	f	G	Göttingen, Leineufer	156	51°31'0.69"N, 9°55'45.55"E	c. 10 m tree, erect	06.05.2023	E. Hörandl
7	S_alba_EH11245	Pure2	m	G	Göttingen, Leineufer	150	51°30'49.63"N, 9°55'34.62"E	c. 20 m tree, erect	06.05.2023	E. Hörandl
8	S_alba_EH11246	Pure2	m	G	Reinhausen, Wendebachtal	186	51°28'14.99"N, 9°57'38.90"E	c. 10 m tree, erect	07.05.2023	E. Hörandl
9	S_alba_EH11247	Pure2	f	G	Reinhausen, Wendebachtal	185	51°28'16.23"N, 9°57'34.63"E	c. 20 m tree, erect	07.05.2023	E. Hörandl
10	S_alba_EH11249	Pure2	m	G	Reinhausen, Wendebachtal	188	51°28'16.88"N, 9°57'34.12"E	c. 10 m tree, erect	07.05.2023	E. Hörandl
11	S_alba_EH11251	Pure2	f	G	Göttingen, Rosdorf	148	51°29'49.30"N, 9°54'52.21"E	c. 20 m tree, erect	13.05.2023	E. Hörandl
12	S_alba_EH11252	Pure2	m	G	Göttingen, Rosdorf	153	51°30'24.31"N, 9°54'53.46"E	c. 30 m tree, erect	02.10.2023	E. Hörandl
13	S_alba_EH11253	Pure2	m	G	Witzenhausen	140	51°21'27.33"N, 9°49'39.87"E	c. 30 m tree, erect	14.05.2023	E. Hörandl & al.
14	S_alba_EH11255	Pure2	f	G	Witzenhausen	133	51°21'28.61"N, 9°49'35.51"E	c. 10 m tree, erect	14.05.2023	E. Hörandl & al.
15	S_alba_EH11256	Pure2	f	G	Witzenhausen	139	51°21'21.44"N, 9°49'31.95"E	c. 5 m tree, erect	14.05.2023	E. Hörandl & al.
16	S_alba_EH11257	Pure2	f	G	Witzenhausen	132	51°21'28.09"N, 9°49'21.24"E	c. 15 m tree, erect	14.05.2023	E. Hörandl & al.
17	S_alba_EH11258	Pure2	f	G	Bad Sooden-Allendorf	149	51°15'35.70"N, 9°58'22.71"E	c. 20 m tree, erect	18.05.2023	E. Hörandl & al.
18	S_alba_EH11259	Pure2	m	G	Bad Sooden-Allendorf	149	51°15'33.43"N, 9°58'14.15"E	c. 20 m tree, erect	18.05.2023	E. Hörandl & al.
19	S_alba_EH11260	Pure2	m	G	Treffurt	176	51° 8'0.31"N, 10°12'59.88"E	c. 20 m tree, erect	21.05.2023	E. Hörandl & al.
20	S_alba_EH11261	Pure2	f	G	Treffurt	176	51° 7'59.19"N, 10°13'0.11"E	c. 30 m tree, erect "excelsa"	21.05.2023	E. Hörandl & al.
21	S_alba_EH11268	Pure2	m	G	Kelbra, am Helme-Stausee	155	51°25'51.87"N, 11° 1'0.93"E	c. 20 m tree, erect	28.05.2023	E. Hörandl & al.
22	S_alba_EH11269	Pure2	f	G	Kelbra, am Helme-Stausee	155	51°25'51.92"N, 11° 0'58.45"E	c. 20 m tree, erect	28.05.2023	E. Hörandl & al.
23	S_alba_EH11270	Pure2	m	G	Seeburg, Seeanger	161	51°34'12.51"N, 10° 7'58.24"E	c. 20 m tree, erect	29.05.2023	E. Hörandl & al.
24	S_alba_EH11271	BackCross2	f	G	Seeburg, Seeanger	163	51°34'0.75"N, 10° 8'20.16"E	c. 20 m tree, erect	29.05.2023	E. Hörandl & al.
25	S_alba_EH11273	Pure2	f	G	Wendebachtal bei Reinhausen	183	51°28'16.07"N, 9°57'35.27"E	c. 10 m tree, erect	04.06.2023	E. Hörandl & al.
26	S_alba_EH11279	Pure2	m	G	Göttingen, Kiessee	150	51°30'46.56"N, 9°55'12.70"E	c. 25 m tree, erect	17.06.2023	E. Hörandl
27	S_alba_EH11280	Pure2	f	G	Göttingen, Kiessee	150	51°30'47.49"N, 9°55'13.83"E	c. 10 m tree, erect	17.06.2023	E. Hörandl
28	S_alba_EH11373	Pure2	m	G	Henschleben	148	51° 8'36.92"N, 10°57'38.45"E	c. 30 m tree, erect	02.04.2024	E. Hörandl, &. al.
29	S_alba_EH11374	Pure2	f	G	Henschleben	148	51° 8'36.92"N, 10°57'38.45"E	c. 30 m tree, erect	02.04.2024	E. Hörandl & al.

30	S_alba_EH11378	Pure2	m	G	Klein Lengden	206	51°29'36.58"N, 9°59'55.11"E	c. 5 m tree, erect	05.04.2024	E. Hörandl
31	S_alba_EH11379	Back-Cross2	f	G	Klein Lengden	206	51°29'36.58"N, 9°59'55.11"E	c. 5 m tree, api-cally pendulous	05.04.2024	E. Hörandl
32	S_alba_EH11383	Pure2	m	G	Göttingen, an der Lutter	173	51°33'21.10"N, 9°56'38.25"E	c. 20 m tree, erect	27.04.2024	E. Hörandl
33	S_alba_EH11384	Pure2	f	G	Göttingen, an der Lutter	173	51°33'19.54"N, 9°56'50.84"E	c. 20 m tree, erect	27.04.2024	E. Hörandl
34	S_alba_EH11385	Pure2	m	G	Göttingen, an der Lutter	186	51°33'21.46"N, 9°57'11.22"E	c. 20 m tree, erect	27.04.2024	E. Hörandl
35	S_alba_EH11386	Pure2	m	G	Kassel, Fuldaaue	140	51°17'35.86"N, 9°30'17.23"E	c. 20 m tree, erect	28.04.2024	E. Hörandl & al.
36	S_alba_EH11387	Pure2	f	G	Kassel, Fuldaaue	136	51°17'52.76"N, 9°30'23.69"E	c. 30 m tree, erect	28.04.2024	E. Hörandl & al.
37	S_alba_EH11388	Pure2	f	G	Kassel, Fuldaaue	140	51°17'48.30"N, 9°30'7.74"E	c. 30 m tree, erect	28.04.2024	E. Hörandl & al.
38	S_alba_EH11389	Pure2	m	G	Kassel, Fuldaaue	140	51°17'44.90"N, 9°30'8.23"E	c. 20 m tree, erect	28.04.2024	E. Hörandl & al.
39	S_alba_EH11390	Pure2	f	G	Kassel, Fuldaaue	140	51°17'14.26"N, 9°29'31.87"E	c. 20 m tree, erect	28.04.2024	E. Hörandl & al.
40	S_alba_EH11391	Pure2	f	G	Hedemünden	268	51°24'18.15"N, 9°48'25.76"E	c. 30 m tree, erect	01.05.2024	E. Hörandl & al.
41	S_alba_EH11392	Pure2	m	G	Hedemünden	268	51°24'17.74"N, 9°48'27.93"E	c. 30 m tree, erect	01.05.2024	E. Hörandl & al.
42	S_alba_EH11396	Pure2	m	G	Dietzenrode - Vat-terode	201	51°18'11.31"N, 10° 1'20.58"E	c. 10 m tree, erect	20.05.2024	E. Hörandl & al.
43	S_alba_x_baby-lonica_EH11230	F1	m+f	G	Witzenhausen	206	51°20'7.99"N, 9°51'12.84"E	c. 10 m tree, cult., pendulous;	23.04.2023/01.05.2024	E. Hörandl
44	S_alba_x_baby-lonica_EH11240	F1	m+f	G	Göttingen, Zentrum	149	51°32'9.84"N, 9°55'57.15"E	c. 20 m tree, cult. pendulous	03.05.2023	E. Hörandl
45	S_alba_x_baby-lonica_EH11241	F1	m	G	Göttingen, Zentrum	162	51°31'46.81"N, 9°55'53.05"E	c. 20 m tree, cult., pendulous	04.05.2023	E. Hörandl
46	S_alba_x_baby-lonica_EH11242_1	F1	m+f	G	Göttingen, Zentrum	162	51°31'48.60"N, 9°55'55.95"E	c. 30 m tree, cult., pendulous	04.05.2023	E. Hörandl
47	S_alba_x_baby-lonica_EH11243	F1	f	G	Göttingen, Stadtfriedhof	171	51°31'48.50"N, 9°54'43.89"E	c. 10 m tree, cult., pendulous	06.05.2023	E. Hörandl
48	S_alba_x_baby-lonica_EH11243_1	F1	f	G	Göttingen, Stadtfriedhof	171	51°31'48.50"N, 9°54'43.89"E	c. 10 m tree, cult., pendulous	04.04.2024/06.05.2023	E. Hörandl
49	S_alba_x_baby-lonica_EH11250	F1	m+f	G	Göttingen, Rein-häuser Landstraße	158	51°30'16.53"N, 9°56'19.39"E	c. 20 m tree, cult., pendulous	07.05.2023	E. Hörandl
50	S_alba_x_baby-lonica_EH11262	F1	m+f	G	Göttingen, Schiller-wiesen	186	51°32'3.49"N, 9°57'10.03"E	c. 15 m tree, cult., pendulous	26.05.2023	E. Hörandl
51	S_alba_x_baby-lonica_EH11263	F1	m+f	G	Klein Lengden	219	51°30'0.01"N, 10° 0'6.29"E	c. 15 m tree, cult., pendulous	27.05.2023/05.04.2024	E. Hörandl
52	S_alba_x_baby-lonica_EH11265	F1	f	G	Klein Lengden	210	51°29'49.93"N, 10° 0'14.40"E	c. 10 m tree, cult., pendulous	27.05.2023	E. Hörandl
53	S_alba_x_baby-lonica_EH11266	F1	f	G	Kelbra, am Helme-Stausee	161	51°25'42.91"N, 11° 1'3.00"E	c. 10 m tree, cult., pendulous	28.05.2023	E. Hörandl & al.
54	S_alba_x_baby-lonica_EH11267	F1	m	G	Kelbra, am Helme-Stausee	155	51°25'50.51"N, 11° 1'6.78"E	c. 15 m tree, cult., pendulous	28.05.2023	E. Hörandl & al.
55	S_alba_x_baby-lonica_EH11272	F1	m	G	Seeburg, Seeanger	163	51°33'52.76"N, 10° 8'17.98"E	c. 20 m tree, cult., pendulous,	29.05.2023	E. Hörandl & al.
56	S_alba_x_baby-lonica_EH11277	F1	m+f	G	Fredelsloh	273	51°44'11.82"N, 9°47'48.07"E	c. 10 m tree, cult., pendulous	11.06.2023/06.04.2024	E. Hörandl & al.
57	S_alba_x_baby-lonica_EH11278	F1	m	G	Göttingen, Geismar	190	51°30'59.75"N, 9°57'16.25"E	c. 10 m tree, cult., pendulous	13.06.2023/15.04.2024	E. Hörandl
58	S_alba_x_baby-lonica_EH11370	F1	m+f	G	Apfelstädter Ried	251	50°52'55.2"N, 10°52'18.1"E	c. 20 m tree, cult., pendulous,	31.03.2024	E. Hörandl & al.
59	S_alba_x_baby-lonica_EH11371	F1	m+f	G	Apfelstädter Ried	251	50°52'55.2"N, 10°52'18.1"E	c. 20 m tree, cult., pendulous,	31.03.2024	E. Hörandl & al.
60	S_alba_x_baby-lonica_EH11372	F1	m	G	Erfurt, Erfurter Gartenausstellung	250	50°58'0.61"N, 11° 0'21.87"E	c. 20 m tree, cult., pendulous,	01.04.2024	E. Hörandl & al.
61	S_alba_x_baby-lonica_EH11376	F1	m+f	G	Göttingen, Kiessee	151	51°30'45.76"N, 9°55'21.43"E	c. 3 m tree, cult., pendulous,	04.04.2024/27.04.2024	E. Hörandl
62	S_alba_x_baby-lonica_EH11377	F1	m+f	G	Göttingen, Stadtfriedhof	156	51°31'49.19"N, 9°54'50.55"E	c. 3 m tree, cult., pendulous,	04.04.2024/27.04.2024	E. Hörandl

63	S_alba_x_babylonica_EH11380	F1	m+f	G	Göttingen, Geismar	222	51°30'59.36"N, 9°57'28.55"E	c. 5 m tree, cult., pendulous,	15.05.2024	E. Hörandl
64	S_alba_x_babylonica_EH11381	F1	m+f	G	Niedernjesa	170	51°28'34.02"N, 9°55'50.51"E	c. 20 m tree, cult., pendulous,	07.04.2024/15.05.2024	E. Hörandl & al.
65	S_alba_x_babylonica_EH11382	F1	m	G	Diemarden	186	51°29'17.06"N, 9°58'48.83"E	c. 4 m tree, cult., pendulous,	13.04.2024/12.05.2025	E. Hörandl
66	S_alba_x_babylonica_EH11393	F1	m+f	G	Hedemünden	239	51°24'6.44"N, 9°47'55.94"E	c. 10 m tree, cult., pendulous,	01.05.2024	E. Hörandl & al.
67	S_alba_x_babylonica_EH11394	F1	m+f	G	Seeburg	165	51°33'56.35"N, 10° 8'31.32"E	c. 20 m tree, cult., pendulous,	10.05.2024	E. Hörandl & al.
68	S_babylonica_622 Italy	Pure1	f	I	Sicily , between Spadafora and Grangiara	80	38° 12'51'' N, 15° 22' 43''E	cult. garden, pendulous	04.04.2023	B. Frajman
69	S_babylonica_EH11264	Pure1	f	G	Klein Lengden	219	51°30'0.41"N, 10° 0'8.42"E	c. 5 m tree, cult., slightly pendulous (tortuose twigs)	05.04.2024/ 27.05.2024	E. Hörandl
70	S_babylonica_S15 China	Pure1	f	Ch	Shanghai Chenshan Botanical Garden	3	31° 4'38.00" N, 121°10'27.00" E	cultivated, pendulous	14.08.2024	Zhiqing Xue, Yuan Wang, Guangnan Gong
71	S_babylonica_S16 China	Pure1	f	Ch	Shanghai Chenshan Botanical Garden	3	31° 4'38.00" N, 121°10'27.00" E	cultivated, pendulous	14.08.2024	Zhiqing Xue, Yuan Wang, Guangnan Gong
72	S_babylonica_S17 China	Pure1	f	Ch	Shanghai Chenshan Botanical Garden	3	31° 4'38.00" N, 121°10'27.00" E	cultivated, pendulous	14.08.2024	Zhiqing Xue, Yuan Wang, Guangnan Gong
73	S_babylonica_S18 China	Pure1	f	Ch	Shanghai Chenshan Botanical Garden	3	31° 4'38.00" N, 121°10'27.00" E	cultivated, pendulous	14.08.2024	Zhiqing Xue, Yuan Wang, Guangnan Gong
74	S_babylonica_S_n Italy	Pure1	f	I	Salerno, Quercioni, Saltavilla Silentina	75	40°31'6.41"N, 15° 6'48.61"E	cultivated, pendulous	10.05.2023	E. del Guacchio

Table S2. Flow cytometric assessment of nuclear DNA levels in *Salix* hybrids. Median relative fluorescence values were calculated and used to determine the ploidy levels of the hybrid samples by comparison with a known standard, considering genome size values reported by Thibault (1998) [1].

	Reported Ploidy	Estimated Ploidy
*S. caprea**	2x (T)	2x
S. alba x babylonica (as *S. xchrysocoma)*	4x (T)	
S. alba × *babylonica* EH11250	-	4x
S. alba × *babylonica* EH11376	-	4x
S. alba × *babylonica* EH11382	-	4x

* The known diploid *S. caprea* was used as a standard. The parental species are also known to be tetraploid: *S. alba* according to Thibault (1998) (T) and *S. babylonica* according to He et al. (2024).

Table S3. ***RADSex*** **comparison summary**. Comparisons shown in green were methodologically and computationally feasible and were evaluated and reported in this study. Comparisons in red were not assessed due to computational and/or methodological limitations of the dataset (e.g., *RADSex* requires two groups for comparison, but *S. babylonica* includes only female samples). Three group comparisons were objectively exploratory analyzed based on several preliminary tests: 1) *S. alba* population (42 samples), comparison of females and males; 2) *S. alba* + *S. alba* × *babylonica* population (67 samples), comparison of males and monoecious individuals (i.e., individuals expressing both male and female phenotypes in the hybrid population). 3) Complete hybrid dataset: *S. alba* + *S. alba* × *babylonica* + *S. babylonica* (total of 74 samples), comparing phenotypic males and monoecious individuals.

	S. alba	***S. alba x babylonica***	***S. babylonica***	**Merged**
	42 Samples (21 Females, 21 Males)	25 Samples (4 Females, 15 Monoecious Plants, 6 Males)	7 Samples (All Females)	74 Samples (32 Females, 15 Monoecious 27 Males)
	Male Heterogamy (XX/XY and XX/XX) *	Mainly both Male and Female Heterogamy **	Female heterogamy (ZZ/ZW and ZZ/Z0) ***	Merged Male, Female Heterogamy and Monoecious
S. alba (S.a)				
S. alba x babylonica (S.axb)				
S. babylonica (S.b)				
**Merged (*S.a* + *Saxb* + *S.b*)**				

* Inferred SDS because *Salix alba* belongs to the Salix clade. ** Males, females, and monoecious individuals were phenotypically identified. *** Female heterogamy is already known in *S. babylonica*.

Reference

1. Thibault, J., Nuclear DNA amount in pure species and hybrid willows (Salix): a flow cytometric investigation. *Can. J. Bot.-Rev. Can. Bot.* **1998**, *76*, 157–165.
